# Optimization of injected ^68^Ga-PSMA activity based on list-mode phantom data and clinical validation

**DOI:** 10.1186/s40658-020-00289-9

**Published:** 2020-04-15

**Authors:** J. Wielaard, J. B. A. Habraken, P. Brinks, J. Lavalaye, R. Boellaard

**Affiliations:** 1 Department of Medical Physics, St. Antonius Hospital, Nieuwegein, Netherlands; 2 Department of Medical Physics, Diakonessenhuis, Utrecht, Netherlands; 3 Department of Nuclear Medicine, St. Antonius Hospital, Nieuwegein, Netherlands; 4 Department of Radiology and Nuclear Medicine, Amsterdam University Medical Centres, Location VUMC, Amsterdam, Netherlands

**Keywords:** PET, ^68^Ga-PSMA, Activity, Optimization

## Abstract

Optimization of injected gallium-68 (^68^Ga) activity for ^68^Ga-prostate-specific membrane antigen positron emission tomography/computed tomography (^68^Ga-PSMA PET/CT) studies is relevant for image quality, radiation protection, and from an economic point of view. However, no clear guidelines are available for ^68^Ga-PSMA studies. Therefore, a phantom study is performed to determine the highest coefficient of variation (COV) acceptable for reliable image interpretation and quantification.

To evaluate image interpretation, the relationship of COV and contrast-to-noise ratio (CNR) was studied. The CNR should remain larger than five, according to the Rose criterion. To evaluate image quantification, the effect of COV on the percentage difference (PD) between quantification results of two studies was analyzed. Comparison was done by calculating the PD of the SUV_max_. The maximum allowable PD_SUVmax_ was set at 20%. The highest COV at which both criteria are still met is defined as COV_max_. Of the NEMA Image Quality phantom, a 20 min/bed (2 bed positions) scan was acquired in list-mode PET (Philips Gemini TF PET/CT). The spheres to background activity ratio was approximately 9:1. To obtain images with different COV, lower activity was mimicked by reconstructions with acquisition times of 10 min/bed to 5 s/bed. Pairs of images were obtained by reconstruction of two non-overlapping parts of list-mode data.

For the 10-mm diameter sphere, a COV of 25% still meets the criteria of CNR_SUVmean_ ≥ 5 and PD_SUVmax_ ≤ 20%. This phantom scan was acquired with an acquisition time of 116 s and a background activity concentration of 0.71 MBq/kg. Translation to a clinical protocol results in a clinical activity regimen of 3.5 MBq/kg min at injection. To verify this activity regimen, 15 patients (6 MBq/kg min) with a total of 22 lesions are included. Additional reconstructions were made to mimic the proposed activity regimen. Based on the CNR_SUVmax_, no lesions were missed with this proposed activity regimen.

For our institution, a clinical activity regimen of 3.5 MBq/kg min at injection is acceptable, which indicates that activity can be reduced by almost 50% compared with the current code of practice. Our proposed method could be used to obtain an objective activity regimen for other PET/CT systems and tracers.

## Introduction

Gallium-68 prostate-specific membrane antigen (^68^Ga-PSMA) positron emission tomography/computed tomography (PET/CT) is a non-invasive diagnostic technique to image prostate cancer. ^68^Ga-PSMA is a promising radioligand [1, 2]. Optimization of injected activity is relevant for image quality, radiation protection (lower radiation dose for the patient), and also from an economic point of view (less activity results in lower costs). The current joint European Association of Nuclear Medicine (EANM)/Society of Nuclear Medicine and Molecular Imaging (SNMMI) procedure guideline recommends an injected dose of 1.8–2.2 MBq/kg ^68^Ga-PSMA 60 min post injection, and an acquisition time of 3–4 min/bed is used [3]. However, this does not take into account the different sensitivities of various PET systems.

Recently, guidelines are published by the European Association of Nuclear Medicine (EANM) for quantitative ^18^F-FDG PET/CT studies [4, 5]: the minimum recommended administered ^18^F-FDG activity for a 75 kg patient, in combination with the minimally intended acquisition time per bed position, should meet two criteria, unbiased standardized uptake value (SUV) recoveries and coefficient of variation (COV) < 15%, as measured in the image quality phantom. Using this COV criterion, it is assumed that clinical image quality will be acceptable when the determined minimum combination of required activity and scan duration is applied. However, this criterion applies to FDG PET/CT studies and it is not evident that this criterion can be translated straightforward to ^68^Ga-PSMA studies.

A general method used for optimization is based on visual inspection by multiple readers. A disadvantage of this method is that it is subjective and dependent on the interpretation of the readers. An objective method for optimization is based on noise equivalent counts [6–8]. This method does not take into account the contrast of the lesions.

Therefore, in this paper, an objective method is proposed to determine the minimum injected activity for clinical ^68^Ga-PSMA imaging studies that is acceptable for reliable image interpretation as well as quantification. Based on phantom data, an acceptable noise level will be set and translated to a clinical activity regimen. Patient scans are used retrospectively to validate the effect of the proposed activity regimen on the image interpretation and quantification.

## Material and methods

A phantom study is performed to determine a maximum acceptable noise level. The maximum noise level will be set at the noise level where image interpretation and quantification is still acceptable. The determined maximum noise level will be translated to a clinical activity regimen for ^68^Ga-PSMA imaging studies. Patient scans are used retrospectively to validate the proposed activity regimen.

### Phantom study

The noise level, which can influence the detectability of the object, is described by the COV:

1 $$ \mathrm{COV}=\frac{{\mathrm{SD}}_{\mathrm{B}}}{S{}_B}\cdot 100\% $$

with SD_*B*_ the standard deviation of the background and S_*B*_ the average activity concentration in the background. The two parameters to analyze image interpretation and quantification, based on which the COV will be set, will be described. The highest COV at which both criteria are still met is defined as COV_max_.

For image interpretation, the contrast-to-noise ratio (CNR) is analyzed. The CNR_SUVmean_ and CNR_SUVmax_ are dimensionless parameters and can be determined based on the SUV_mean_ or the SUV_max_, respectively:

2 $$ {\mathrm{CNR}}_{\mathrm{SUVmean},\mathrm{j}}=\frac{S_{mean,j}-{S}_B}{{\mathrm{SD}}_{\mathrm{B}}} $$

3 $$ {\mathrm{CNR}}_{\mathrm{SUVmax},\mathrm{j}}=\frac{S_{\max, j}-{S}_B}{{\mathrm{SD}}_{\mathrm{B}}} $$

with *S*_mean,*j*_ and *S*_max,*j*_ the average and maximum activity concentration for a region-of-interest (ROI) *j*, *S*_*B*_ the background activity concentration, and SD_*B*_ the standard deviation of the background activity concentration. The CNR_SUVmean_ will be used for optimization. A lower CNR is expected with higher noise levels. In this study, image interpretation is assumed to be acceptable if the object fulfills the Rose criterion [9]: the CNR needs to be equal to or larger than 5 to be detectable.

For image quantification, a new parameter is introduced: namely, the percentage difference (PD) between quantitative results of two studies that are as equal as possible. These studies are two acquisitions of the same phantom, in the same position, with the same acquisition and reconstruction parameters. The only difference between these two studies is the noise statistics. The PD gives information about the extent at which noise affects the quantitative results. Increasing noise will affect the activity concentration in such extent that the signal becomes unreliable. The PD is defined as follows:

4 $$ {\mathrm{PD}}_{\mathrm{SUV}\max }=\left|\frac{S_{\max, j,\mathrm{P}1}-{S}_{\max, j,\mathrm{P}2}}{\frac{1}{2}\left({S}_{\max, j,\mathrm{P}1}+{S}_{\max, j,\mathrm{P}2}\right)}\right|\cdot 100\% $$

5 $$ {\mathrm{PD}}_{\mathrm{SUV}\max }=\left|\frac{S_{\mathrm{mean},j,\mathrm{P}1}-{S}_{\mathrm{mean},j,\mathrm{P}2}}{\frac{1}{2}\left({S}_{\mathrm{mean},j,\mathrm{P}1}+{S}_{\mathrm{mean},j,\mathrm{P}2}\right)}\right|\cdot 100\% $$

with *S*_mean,*j*_ and *S*_max,*j*_ the average and maximum activity concentration in a region-of-interest *j*, for two studies P1 and P2. The PD_SUVmax_ will be used for optimization. A larger difference is expected with higher noise levels. The threshold for the PD_SUVmax_ is based on thresholds regarding tumor response. A 20% change in uptake is considered to reflect true changes in metabolism [10, 11]. Therefore, image quantification is assumed to be acceptable at a PD_SUVmax_ smaller than 20%.

To assess the SUV recoveries, the recovery coefficients (RC) will be visualized. The RC of a ROI *j* is the ratio of the apparent activity concentration to the true activity concentration and is defined as follows:

6 $$ {\mathrm{RC}}_j=\frac{S_{j,\mathrm{measured}}/{S}_{B, measured}}{S_{j. true}/{S}_{B, true}} $$

#### Phantom data

All images used for this study were acquired with a Philips Gemini TF PET/CT system (Philips Healthcare, Andover, MA). PET reconstructions were made using scanner’s default ordered subset expectation maximization (OSEM) reconstruction algorithm with 33 subsets, 3 iterations, matrix size of 144 × 144, and voxels of 4 × 4 × 4 mm. No Gaussian filter is applied. The reconstruction corrects for geometrical response and detector efficiency (normalization), random coincidences, scatter, and attenuation. Data were stored in list-mode, to be able to reconstruct different acquisition times. All list-mode reconstructions are decay-corrected to the start time of the acquisition.

A dataset of the NEMA NU2-2001 Image Quality phantom (IQ phantom) was created. The six spheres (10, 13, 17, 22, 28, and 37 mm diameter) of the IQ phantom were filled with a known concentration ^68^Ga. The NEMA lung insert was situated at the center of the phantom. The IQ phantom was filled with activity concentrations of the spheres and background compartment of 6.48 kBq/mL and 0.71 kBq/mL, respectively (ratio of approximately 9:1), at acquisition time. A 20 min/bed scan was acquired in list-mode PET (5,284,119 counts in the central slice through the spheres). A CT scan was performed for attenuation correction. To analyze the influence of the COV on image interpretation as well as quantification, additional list-mode reconstructions with different COV values are required.

For image interpretation analysis, subsampled reconstructions were made using the list-mode data of the 20-in/bed scan. Nineteen reconstructions were made, of which acquisition times varied from 10 min/bed to 5 s/bed, referred to as reconstructions part 1 (P1). These P1 reconstructions were used for evaluating image interpretation.

For image quantification analysis, pairs of images were obtained by reconstruction of two non-overlapping parts of list-mode data. The reconstructions of the second part of the list-mode data are further referred to as part 2 (P2) reconstructions. The results of the PD_SUVmax_ parameter are presented against the COV of the background from the P1 reconstructions.

#### Image analysis

For analysis, ROIs were drawn as circular areas with diameters equal to the physical inner diameters of the spheres. A script was used to place ROIs automatically on the 20 min/bed scan, in which the position of the spheres could be determined most accurately. Twelve ROIs with a 37-mm diameter were drawn in the central slice, as well as in the slices − 20, − 10, + 10, and + 20 mm from the central slice. The average of all 60 ROIs was used as the background value *S*_*B*_. Coordinates of all ROIs were kept equal for all images.

Higher CNRs are expected with increasing sphere diameter; therefore, only the 10-mm diameter sphere of the IQ phantom is analyzed.

### Clinical validation

#### Translation to clinical regimen

To validate the effect of the proposed activity regimen on clinical data, the COV_max_ needs to be translated to a clinical regimen. A minimum clinical acquisition time per bed position can be derived, by comparing the activity concentration of the phantom experiment with the activity concentration in a patient [12]. The following formula is used:

7 $$ {T}_{\mathrm{min}}={\left(\frac{a}{{\mathrm{COV}}_{\mathrm{max}}}\right)}^{\frac{1}{b}}\cdot \left(\frac{\left[{B}_{true}\right]}{\left[{B}_{\mathrm{reference}}\right]}\right) $$

The parameters *a* and *b* will be obtained using a power-law fit through the obtained phantom data: COV = *a* T^−*b*^. [*B*_true_] represents the measured activity concentration in the background of the phantom. [*B*_reference_] represents the background activity concentration in a patient with a reference body weight of 75 kg. In our clinical practice, a patient of 75 kg receives a ^68^Ga activity of 112.5 MBq 60 min post injection (1.5 MBq/kg). Based on the assumption of a homogeneous distribution of the radionuclide throughout the background and by taking into account decay between injection and acquisition (60 min) and urine clearance (27%), this activity concentration equals 0.59 kBq/mL. Using formula (7), the COV_max_ results in a minimum scan time per bed position. This can be translated to a linear clinical activity regimen [MBq/kg min].

#### Clinical data

To validate the effect of the proposed activity regimen on the image interpretation and quantification, patient scans were used retrospectively. Fifteen male patients (71 ± 6 years, 85 ± 13 kg) underwent a ^68^Ga-PSMA PET/CT scan from the top of the head to the mid-thigh, 60 min after intravenous injection. Patients were injected according to our standard protocol: 1.5 MBq/kg, 4 min/bed, which equals 6 MBq/kg min. All images were acquired on the same PET/CT system and reconstructed with the same parameters as the phantom study. Both examinations, for baseline and staging purposes, were allowed. This study was conducted according to the Declaration of Helsinki [13].

Additional reconstructions were made to mimic the proposed activity regimen. Similar to the phantom study, the P1 reconstructions were made for image interpretation by reconstruction of the list-mode data with acquisition times of 180, 140, 120, 90, 60, and 30 s/bed. The P2 reconstructions with acquisition times of 120, 90, 60, and 30 s/bed were obtained for image quantification. P2 reconstructions larger than half of the acquired data were not allowed, in order to avoid overlap of counts.

#### Image analysis

Lesions were indicated on retrospective data by one nuclear physician as done in clinical routine. ROIs representing the lesions were drawn on the initial scan using the software package Hermes Hybrid Viewer version 2.6 (Hermes Medical Solutions, Stockholm). Lesions are delineated using the isocontour of 50% of the SUV_max_ of the lesions (A50). As a reference, the surrounding background activity was determined by calculating the mean of 3 ROIs in the neighbouring tissue. Lesion and background ROIs were copied to all reconstructions.

#### Validation

The validation of the proposed activity regimen is based on objective measurements (no human observer is involved). On each reconstruction, the CNR_SUVmean_ and CNR_SUVmax_ of each lesion is measured. A lesion is defined as being missed if the CNR_SUVmean_ is smaller than 5 or if the PD_SUVmax_ is larger than 20%.

## Results

### Phantom results

**Fig. 1:**
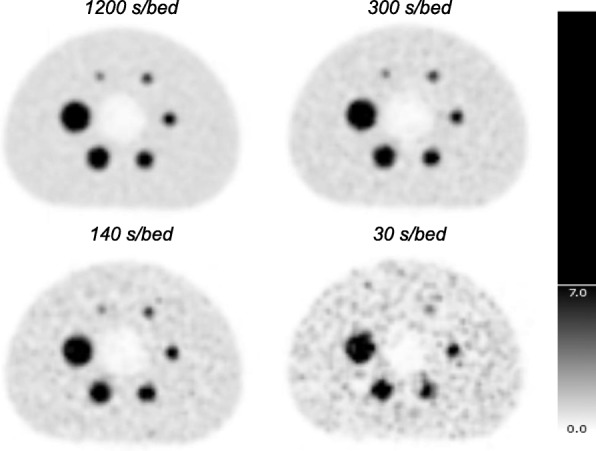
Visualization of reconstructions with shorter acquisition times and therefore lower statistics

Shorter acquisition times result in increasing noise, i.e., higher COV values. Figure 1 shows a series of reconstructions in which the acquisition time decreases.

**Fig. 2:**
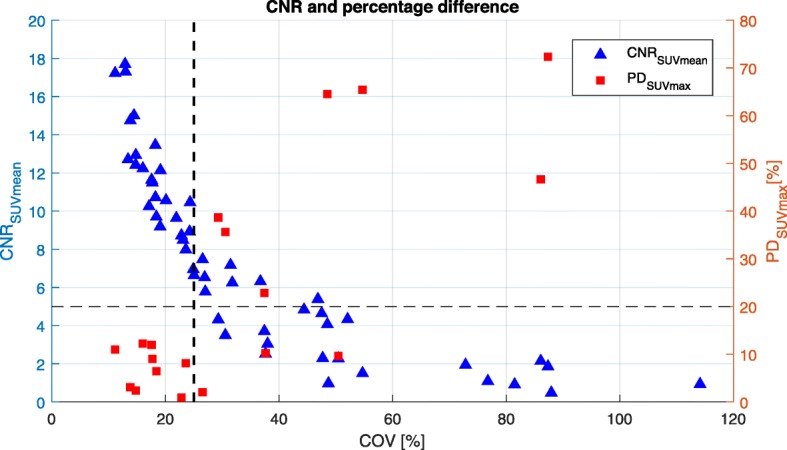
The coefficient of variation plotted against the CNR_SUVmean_ (left *y*-axes) and the percentage difference (right *y*-axis), determined for the 10-mm diameter sphere of the IQ phantom. For the CNR, the reconstructions of P1 and P2 are included, together with three additional non-overlapping reconstructions for the acquisition times of 240, 140, 100, 30, and 10 s/bed. The PD_SUVmax_ is plotted against the COV of P1 reconstructions. The COV_max_ = 25% (vertical dotted line), CNR = 5, and PD = 20% (horizontal dotted line) are indicated

The CNR_SUVmean_ decreases and the PD_SUVmax_ rises while the COV increases. For high COV, the PD is susceptible to noise; therefore, a higher deviation is present. Results for the 10-mm diameter sphere are presented against the COV, see Fig. 2. At a COV of 27%, the CNR_SUVmean_ of the 10-mm diameter sphere drops below 5, and the 20% threshold for the PD_SUVmax_ is exceeded. Therefore, a COV of 25% is set as COV_max_.

**Fig. 3:**
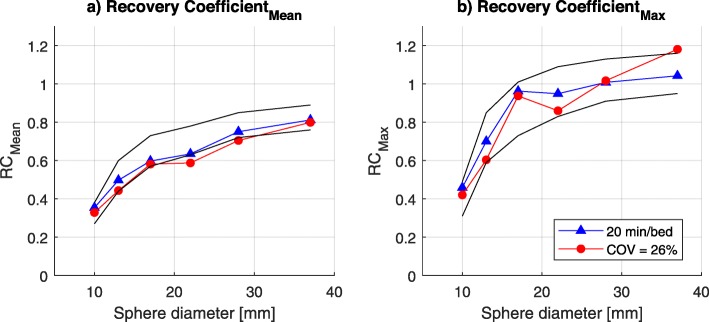
**a** RC_mean_ and **b** RC_max_ values for all spheres for the PET reconstructions of ^68^Ga scan of 20 min/bed and COV = 26% (100 s/bed), compared to EARL minimum and maximum accreditation specifications for ^18^F [14]

The RCs of the COV = 26% scan (phantom scan nearest to COV_max_) were compared to the RCs of the 20 min/bed ^68^Ga PET/CT scan. The mean and maximum RCs are presented in Fig. 3, together with the ^18^F EARL limits. As expected, both scans show mean RCs at the lower limit of the current ^18^F EARL RC limits. This is previously observed in other centers as well and is likely explained by the larger positron range of ^68^Ga compared to ^18^F [14]. At the maximum RCs, an upward bias is seen for the largest sphere for the COV = 26% scan compared to the 20 min/bed scan.

**Fig. 4:**
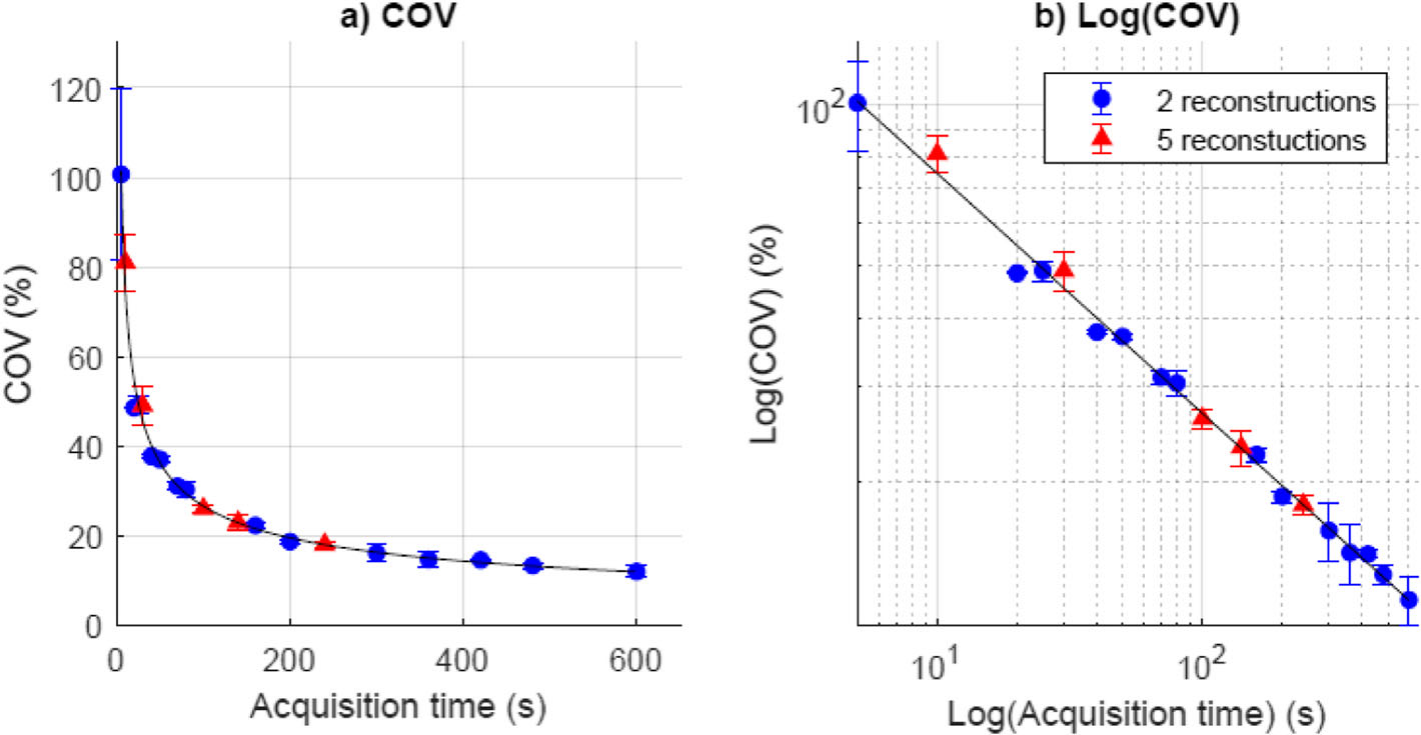
Coefficient of variation (COV) in the phantom background compartment of both, P1 and P2 reconstructions at different acquisition times, in graphs with **a** standard scale and **b** log-log scale. A power-law fit resulted in COV = 208 *T*^−0.446^. The coefficient of determination *r*^2^ equals 0.99, which indicates a good fit. Three additional non-overlapping reconstructions were included for five acquisition times (240, 140, 100, 30, and 10 s/bed) to obtain a measure of error

In Fig. 4, the COV is presented against the acquisition time. A power-law fit allows us to interpolate and to find the minimum required acquisition time to achieve a COV of 25% [12]. For the phantom, a minimum acquisition time of 116 s is required to achieve a noise level of 25%.

To translate the result of the phantom experiment to a clinical protocol, formula (7) is used to compare the activity concentration in the patient to the activity concentration in the phantom. The power-law fit results in parameters *a* = 208 and *b* = 0.446. The injected background activity concentration in the phantom equals 0.71 kBq/mL, and the reference activity concentration is assumed to be 0.59 kBq/mL. For a COV of 25%, a minimum acquisition time per bed of 140 s is needed. For a regimen of 1.5 MBq/kg, this results in a clinical activity regimen of 3.5 MBq/kg min at injection.

### Clinical results

**Fig. 5:**
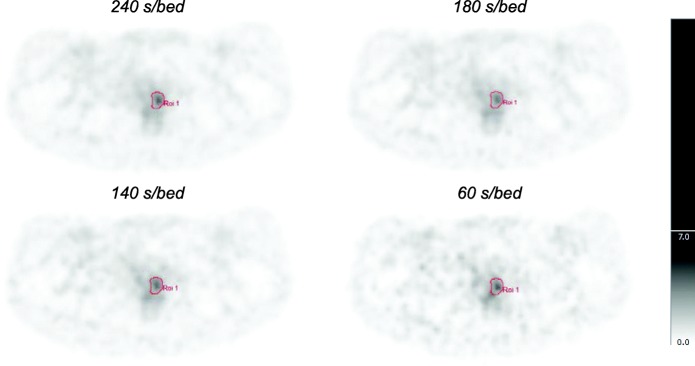
Visualization of reconstructions of a lesion in the prostate, reconstructed with shorter acquisition times

Patient scans are analyzed according to the proposed activity regimen of 3.5 MBq/kg min, which corresponds to 140 s/bed for patients injected according to our current clinical activity regimen. Figure 5 shows a series of reconstructions with acquisition times of 240, 180, 140, and 60 s/bed.

**Fig. 6:**
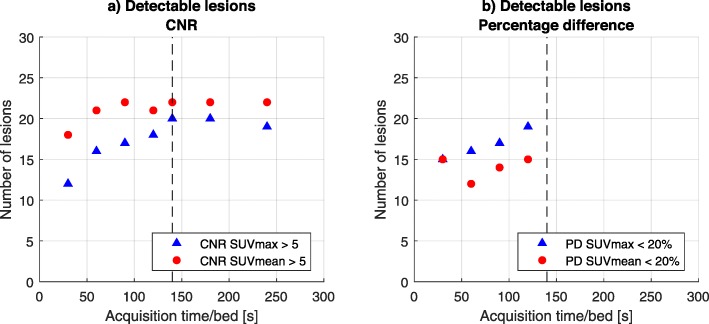
Detected lesions based on **a** CNR_SUVmean_ and CNR_SUVmax_ > 5 at the subsampled list-mode reconstructions with acquisition times (240, 180, 120, 90, 60, and 30 s/bed) and **b** PD_SUVmean_ and PD_SUVmax_ < 20% at the subsampled list-mode reconstructions with acquisition times (120, 90, 60, and 30 s/bed). In both graphs, the proposed acquisition time 140 s/bed is indicated (vertical dotted line)

The number of lesions that were detected at different acquisition times, considering CNR and PD, are shown in Fig. 6.

For CNR_SUVmean_, at an acquisition time of 240 s/bed, 19/22 lesions are detectable, compared to 20/22 for 140 s/bed. For CNR_SUVmax_, 22/22 lesions are detectable for an acquisition time of 240 and 140 s/bed. Regarding quantification, 19/22 and 15/22 of the lesions have a PD of less than 20% at an acquisition time of 120 s/bed based on the PD_SUVmean_ and PD_SUVmax_, respectively. No PD data can be obtained for the recommended 140 s/bed acquisition time, because the proposed acquisition time corresponding to the proposed activity regimen is more than half of the acquired data. Therefore, it is not known how many lesions have a PD of less than 20% at 140 s/bed according to this parameter.

## Discussion

In this paper, a method is presented to obtain a COV criterion for ^68^Ga-PSMA imaging studies. Results suggest that a COV of less than 25% is needed in order to keep image interpretation and quantification within acceptable limits.

The COV_max_ is determined by evaluation of the CNR_SUVmean_ and the PD_SUVmax_ of the spheres of the IQ phantom. The formula of the CNR_SUVmean_ should be corrected for the number of pixels of the ROI [15]. This correction is not applied in this study: a ROI size of 1 pixel is assumed. Therefore, the formula is used conservative. Although the SUV_peak_ is a more robust parameter [16], we focused on the most general used SUV_max_ and SUV_mean_, following the recommendations of the European Organization for Research and Treatment of Cancer (EORTC) [17] and the Cancer Imaging Program of the National Cancer Institute [18].

In this study, we assume that subsampled list-mode reconstructions are equal to shorter acquisition times. However, the random rate of the list-mode reconstructions for short acquisition times is higher compared to acquired data. Acquired data with short acquisition times will therefore slightly be of better image quality than the subsampled list-mode data. Our method will thus provide an activity regimen that guarantees the desired image quality.

The phantom scan with a COV_max_ of 26% is used to visualize the RCs. The RC_mean_ nearly reach EARL performance specifications for ^18^F, as expected [14]. The RC_max_ is outside the limits for the largest sphere. It seemed unexpected that the RC_max_ values of this scan are lower than the RC_max_ of the 20 min/bed scan. Upward bias is expected with low count statistics, because of a higher chance that variation will lead to a higher maximum value [19–21]. This effect increases with larger regions. The RC curve is noisy as no Gaussian filter is applied. A noisy RC curve also indicates that the injected activity indeed is at the lower limit. We need to take into account that treatment response assessment can be done only if the reconstruction protocol meets the EARL requirements [22].

The COV_max_ is determined based on the smallest sphere of the IQ phantom (10-mm diameter) and one sphere to background activity ratio (approximately 9:1). Therefore, the proposed activity regimen is optimized for lesions ≥ 10 mm. However, since detectability of lesions indeed depends on the uptake and size of lesions, further research is required for different uptake ratios and lesion sizes [23].

A COV_max_ of 25% results in a proposed activity regimen of 3.5 MBq/kg min at injection. This activity regimen is derived from phantom data and is based on the assumption of a background activity concentration of a reference patient. However, the activity concentration in patients varies. Therefore, the result of the phantom study is validated with patient data.

For the clinical validation, multiple reconstructions of the same patient were analyzed. Visual inspection of a human observer was therefore not possible, since it would cause bias while viewing the scans. For that reason, an objective method of measuring the CNR_SUVmean_ and CNR_SUVmax_ is chosen. This method has a disadvantage, because some lesions detected by the nuclear physician on the 240 s/bed scan did not fulfil the Rose criterion of CNR_SUVmean_ ≥ 5. Therefore, based on the optimization parameter, not all lesions were assumed to be detectable on the 240-s/bed scan. The lowest CNR_SUVmean_ detected by the physician was CNR_SUVmean_ of 3.82. Nevertheless, all lesions maintain a CNR_SUVmax_ larger than 5. For the CNR_SUVmean_, it is unexpected that the number of detectable lesions is lower for 240 s/bed compared to 180 s/bed. However, the CNR_SUVmean_ values were 4.99 and 5.01, respectively. For the PD criterion, it is not known how many lesions have a PD of less than 20% at 140 s/bed, since the PD value cannot be calculated for this dataset due to the fact that the 240-s/bed scan cannot be split into two sets of 140 s without overlap.

ROIs representing lesions were delineated based on the A50 on the 240-s/bed scan. The same ROIs are used to evaluate the lesions on the additional list-mode reconstructions. This choice influences the CNR values, because the CNR is underestimated on these additional list-mode reconstructions. This assumption is part of our approach with which we aim to optimize the dosage regimen but want to prevent a dose that hampers image interpretation or quantification.

For lesion detectability, a ROC analysis could be considered. However, a ROC analysis is not applicable on this dataset since it requires a threshold parameter and the evaluation of false positives and false negatives.

In this study, the proposed regimen is supported by clinical images using a linear activity regimen. However, it is known that a linear relationship with patient-weight leads to a relative poor image quality for heavier patients [24]. Therefore, based on our findings, a new activity regimen shall be proposed which also takes into account the non-linear relationship of the body weight on the injected activity [12]. Evaluation of the proposed activity regimen shall be done and will include analysis of detection performance by visual inspection of nuclear physicians, including false positives.

As more sensitive PET/CT systems and different reconstruction protocols are available, small lesion detectability will improve [25–27]. Reduction of acquisition time can be applied without compromising the noise level. For more sensitive PET/CT systems, the COV_max_ < 25% criterion will result in a different activity regimen for ^68^Ga-PSMA studies. To compare different systems and to take into account the system performance, the noise equivalent count rate (NECR) is a valuable complimentary metric.

For our PET/CT system (Philips Gemini TF PET/CT system), a clinical activity regimen of 3.5 MBq/kg min at injection is recommended, which indicates that activity can be reduced by almost 50% for diagnostic readings of scans. These findings seem to be in contrast with a previous study, which concludes that reduction of injected activity is not feasible [28]. However, the study of Rauscher et al. is based on subjective image quality and lesion contrast scores. It is likely that lesion contrast scores will decrease with lower injected activity, but a subjective measure does not indicate whether the lesion contrast is sufficient to be detectable. In our study, we used an objective detectability threshold to evaluate the possible reduction of injected activity.

Using our method, we have found that a COV lower than 25% is sufficient for correct image interpretation and quantification of ^68^Ga-PSMA studies. However, extensive research is needed to evaluate whether a COVmax < 25% can be a general criterion for ^68^Ga-PSMA studies.

## Conclusion

In this study, the minimally required amount of injected activity of ^68^Ga-PSMA was optimized without compromising image interpretation and image quantification. A method is presented to determine a noise level criterion for ^68^Ga-PSMA imaging studies from phantom data, which can be translated into a clinical activity regimen. Our method suggests that a maximum noise level of 25% is acceptable for correct image interpretation and quantification of ^68^Ga-PSMA studies. The method to determine a noise level criterion for ^68^Ga-PSMA imaging studies was proposed and validated using PET/CT patient scans.

## Data Availability

The datasets used and/or analyzed during the current study are available from the corresponding author on reasonable request.

## Abbreviations

^18^F: Fluor-18
FDG: Fluordeoxyglucose
^68^Ga: Gallium-68
EANM: European Association of Nuclear Medicine
EARL: EANM Research Ltd.
CNR: Contrast-to-noise ratio
CT: Computed tomography
COV: Coefficient of variation
IQ phantom: NEMA NU2-2001 image quality phantom
P1: Reconstructions used for evaluating image interpretation
P2: Additional reconstructions used for evaluating image quantification
PET: Positron emission tomography
PET/CT: Computed tomography/positron emission tomography
PSMA: Prostate-specific membrane antigen
RC: Recovery coefficient
ROI: Region of interest
SUV: Standardized uptake value
